# PortEco: a resource for exploring bacterial biology through high-throughput data and analysis tools

**DOI:** 10.1093/nar/gku902

**Published:** 2014-10-11

**Authors:** J.C. Hu, G. Sherlock, D.A. Siegele, S.A. Aleksander, C.A. Ball, J. Demeter, S. Gouni, T.A. Holland, P.D. Karp, J.E. Lewis, N.M. Liles, B.K. McIntosh, H. Mi, A. Muruganujan, F. Wymore, P.D. Thomas

Nucl. Acids Res. (2014) Jan;42(Database issue):D677–84. doi: 10.1093/nar/gkt1203.

Author Tomer Altman was accidently omitted from the list of authors of the above article. The full list of authors and affiliations is provided below.

The authors apologize to the readers and publisher for any inconvenience this may have caused.

James C. Hu^1^, Gavin Sherlock^2^, Deborah A. Siegele^3^, Suzanne A. Aleksander^1^, **Tomer Altman^4^**, Catherine A. Ball^2^, Janos Demeter^2^, Sushanth Gouni^1^, Timothy A. Holland^4^, Peter D. Karp^4^, John E. Lewis^1^, Nathan M. Liles^1^, Brenley K. McIntosh^1^, Huaiyu Mi^5^, Anushya Muruganujan^5^, Farrell Wymore^2^ and Paul D. Thomas^5^

^1^Department of Biochemistry and Biophysics, Texas A&M University, College Station, TX 77843, USA

^2^Department of Genetics, Stanford University, Stanford, CA 94305, USA

^3^Department of Biology, Texas A&M University, College Station, TX, 77843, USA

^4^Artificial Intelligence Center, SRI International, Menlo Park, CA 94025, USA

^5^Department of Preventive Medicine, University of Southern California, Los Angeles, CA 90089, USA

Present address: Tomer Altman, Biomedical Informatics Program, Stanford University, Stanford, CA 94305, USA

